# The Impact of Social Support on the Health of the Rural Elderly in China

**DOI:** 10.3390/ijerph17062004

**Published:** 2020-03-18

**Authors:** Yunli Bai, Fang Bian, Linxiu Zhang, Yueming Cao

**Affiliations:** 1 Key Laboratory of Ecosystem Network Observation and Modeling, Institute of Geographic Sciences and Natural Resources Research, Chinese Academy of Sciences, Beijing 100101, China; lxzhang.ccap@igsnrr.ac.cn (L.Z.); caoym.17b@igsnrr.ac.cn (Y.C.); 2 International Ecosystem Management Partnership, UN-Environment, Beijing 100101, China; 3 Capital Airport Sub-district Office, Chaoyang District, Beijing 100621, China; bianfang15@mails.ucas.ac.cn; 4 The Department of yueming Cao is Key Laboratory of Ecosystem Network Observation and Modeling, Institute of Geographic Sciences and Natural Resources Research, University of Chinese Academy of Sciences, Beijing 100101, China

**Keywords:** China, rural elderly, social support, physical health, depression

## Abstract

With the dramatic trend of global aging, the physical and mental health of the rural elderly has attracted significant attention. Social support plays an important role in improving the health of the elderly. However, assessing the impact of social support on the physical and mental health of the elderly is challenging in rural China. This paper analyzes the impact of social support on the physical and mental health of the Chinese rural elderly based on data collected from households and village cadres. Probit, Oprobit, and ordinary least squares (OLS) are used to estimate these effects. The results show that 24.3% of the rural elderly are in bad physical health, and 32.9% of them are depressed. Physical and mental health is worse among the female elderly and among those who are in western provinces. Having pensions, taking care of grandchildren, and communicating with children by telephone are shown to significantly improve the mental health of the rural elderly. The government could promote the mental health of the rural elderly by improving public health services, increasing pensions, providing free mobile phones to elderly people in poverty, and advocating that the younger generation provide emotional support.

## 1. Introduction

The aging population presents a global challenge for the world’s social and economic development. The proportion of the world’s population above the age of 65 reached 8.9% in 2018 [1]. The World Health Organization (WHO) reported that the number of people over 60 years old will exceed the number of children under 5 years old by 2020. The proportion of the world’s population over 60 will nearly double from 12% to 22% between 2015 and 2050 [2]. Although population aging started in high-income countries (for example, 30% of Japan’s population is now over 60), the biggest changes are now being seen in low- and middle-income countries [2]. All countries encounter major challenges and must ensure that their health and social systems are prepared to cope with the potential consequences of this demographic shift.

With the rapid improvement of people’s living standards and the implementation of family planning policies, China’s aging population situation is becoming increasingly severe. According to the National Bureau of Statistics (NBS), the proportion of the elderly population over 65 within the total population of China increased from 6.96% in 2000 to 10.8% in 2016 [3]. It is expected that, by 2020, the number of elderly people over 60 will increase to about 255 million, accounting for 17.8% of the total population, and the old-age dependency ratio will increase to 28% [4]. It has taken France almost 150 years to adjust to the increase in the proportion of its population over 60 from 10% to 20%. However, China has had little more than 20 years to adapt to the same changes. This aging trend is more obvious in rural areas, since young laborers flow into cities [5,6,7,8,9].

While the aging process is accelerating, the health outlook for the elderly is not optimistic, especially in rural areas. From a global perspective, there is little evidence that the elderly of today are healthier than their parents [2]. According to the report of the National Working Committee on Aging in China (NWCA), the health status of 27.7% of the rural elderly was self-reported as “good” compared with the national average of 32.8%. The elderly in rural areas reported unhappiness, low life satisfaction, and more prominent mental loneliness than those in cities [10,11,12,13]. If an elderly person is able to spend more years healthy or living in a supportive environment, their ability to engage in activities will be almost indistinguishable from that of a young person. If these years are spent largely in physical and mental decline, this cognitive downturn will have an adverse effect on both the elderly and society.

In order to build a healthy China and improve people’s health, the State Council issued the “Outline of the ‘Healthy China 2030′ Plan” in 2016 [14]. This outline notes that it is necessary to use fairness and justice as strategic principles; it also highlights the health of key populations, such as the elderly, focuses on rural and grass-roots units, and gradually reduces the differences in basic health services and health status between urban and rural areas, regions, and populations to achieve universal health and the promotion of social equity. The key to narrowing the gap in the health of the elderly in urban and rural areas is to improve the health of the rural elderly. Therefore, it is important to fully understand the health and its determinants among the rural elderly.

The effect of social support on the health of the elderly has been a strong focus for scholars. In America, social support for the elderly was shown to retard further deterioration of their health [15]. The relationship between social support and depression is significant, even controlling for negative affectivity among the American elderly [16]. Based on assessing a large amount of research, it is shown that supportive relationships protect us from a multitude of mental health problems, but also that the absence of supportive relationships increases the risk of dying from various diseases [17]. Social support is a mediator between interpersonal sensitivity symptoms and quality of life, and can improve the health of the elderly [18]. Other scholars offer similar conclusions, noting that social support is positively correlated with the health among the elderly in developing countries [19,20].

The scholars also focused on the relationship between social support and the health of the elderly in China. There are two types of studies on this topic in China. Some scholars have used the Social Support Rating Scale developed by Shuiyuan Xiao in 1987 to measure social support, and analyzed the impact of social support on the health of certain types of urban residents, such as retirees, nurses, and immigrants [21,22,23]. Other scholars have analyzed specific types of social support on the health of the elderly using a sample of urban elderly or both urban and rural elderly. This specific social support involves pensions, taking care of grandchildren, and financial support from children [24,25,26,27].

Despite compelling evidence showing that social support is associated with the health of the elderly, there are still some gaps in this topic for the Chinese rural elderly in the current context. First, most studies focus on social support only from one or a few dimensions, such as pensions, taking care of grandchildren, or financial support from one’s children. However, social support is a system concept that involves the government, community, family, and social organizations, and is not fully defined by one or a few variables. Second, previous studies on this topic in China have used a sample of urban elderly or both urban and rural elderly. However, it is well known that there are huge gaps between the development and public service provisions of urban and rural areas in China. Social support for the rural elderly differs greatly from that of urban residents. Therefore, it is important to study the effects of social support in rural areas on the physical and mental health of the rural elderly. Further, the correlation of physical health and mental health has been neglected in previous studies.

This study uses nationally representative survey data collected by the authors to update information on the physical and mental health among the rural elderly in 2015 and attempts to systematically understand the effects of social support on the physical and mental health among the rural elderly by measuring their social support from the perspective of the household, community public service, and national policies. In addition, this study explores the correlation between physical health and mental health among the rural elderly. There is no precise definition for “elderly people.” Most developed countries use 65 years or above as the standard age for the elderly, while the World Health Organization uses 50 years or above as the standard for the elderly in Africa. According to China’s current status of socio-economic development and existing research, this article considers the age of 60 and over as the standard for the “elderly” in rural areas of China.

The remaining sections of this study are as follows. Section 2 introduces the framework and hypothesis of this study. Section 3 features the approach of this study, including sampling, data collection, and definitions of the variables. Section 4 shows the descriptive results. Section 5 presents the regression results. Section 6 provides conclusions and implications.

## 2. Framework and Hypotheses

### 2.1. Social Support in Chinese Rural Areas

The concept and operationalization of social support are changing. In the 1980s, the concept and operationalization of social support were mainly organized into three broad categories: social embeddedness, perceived social support, and enacted support [28]. From the perspective of the function of social support, there was esteem support, information support, social companionship, and instrument support [29], as well as quality of support, received support, emotional support, and structural support [17]. The social support in earlier studies was measured using the Social Support Rating Scale. With the development of economy and society, the operationalization and functions of social support became too complicated to distinguish and measure. Consistent with recent studies on social support in China, we classified the social support in rural China into formal and informal social support.

The principals of formal social support involve formal organizations at all levels of government, as well as institutions, enterprises, and communities. Social support provides social pension systems, medical security systems, and policies to benefit the elderly. In China, the government began to carry out the New Rural Pension Scheme (NRPS) and the New Rural Cooperative Medical (NRCM) in the first decade of this century. The NRPS seeks to improve the livelihood of the rural elderly by paying pensions after continuously affording endowment insurance for 15 years before the age of 60. In rural China, residents can buy different types of pensions. NRPS and commercial pension insurance is common. The premium for NRPS is far lower than that for a commercial pension because it is subsidized by the central and local governments. Consequently, there are fewer pensions from NRPS than commercial pensions. The NRCM aims to alleviate the pressure from medical expenditures by providing an account. There are also various types of social support in the village that provide public services, such as elderly activity centers, which offer entertainment for the elderly. Informal social support usually comes from family members, neighbors, friends, and peers, who are important sources of social support [30]. They provide emotional, behavioral, and informational support [24,25,30,31]. Due to the fact that pensions are far lower in rural areas than in urban areas, the children of the elderly are the main providers of economic support in China. Furthermore, family happiness is very important for grandparents in China, and many left-behind children in rural areas are cared for by their grandparents. Taking care of grandchildren gradually becomes an important factor that affects the health of the rural elderly.

### 2.2. Literature Review

Previous studies have shown that formal and informal support have a different impact on the physical and mental health of the elderly. The self-reported health for elderly citizens with a pension is better than that for those without any pension, and the elderly who communicate more with their families have better mental health than those who communicate with others [32,33]. The financial support and daily care of the rural elderly provided by their children have a positive impact on the elderly’s physical and mental health [27,31]. However, some studies found that financial support from their children did not significantly improve the mental health of the elderly; but a subsidy transferred by the government and village collective did promote their mental health [27]. Support from children and participation in social activities had significant effects on the health of the elderly [34]. In addition, a large number of studies have shown that age, gender, and marital status are important demographic factors that affect the health of the elderly [35,36,37,38]. Socioeconomic status, such as income and education, also has a positive impact on the health of the elderly [39,40,41]. The effects of social support may vary by characteristics such as age, sex, socioeconomic status, cultural setting, and disease [17,42,43].

Although many scholars have conducted research on the health issues of all the elderly in recent years in China, their research results did not provide targeted suggestions for the rural elderly [11,27,44]. First, previous studies on this topic in China used a sample of urban elderly or both urban and rural elderly in individual provinces or even a single district, none of which provide national representativeness [42,43,44,45,46,47]. Although a few studies used a large amount of sample survey data with national representation, the data were out of date, which makes it difficult to determine the current health of the rural elderly. Second, most studies focus on social support from only one or a few dimensions, such as pensions, taking care of grandchildren, or financial support from children [24,25,26,27]. However, social support is a system concept that involves the government, community, family, and social organizations and is not fully defined by one or a few variables. Third, there is a lack of measurement for social support at the community level in the literature, although previous studies found that the provision of social service facilities for the elderly had a significant impact on their health [48]. Fourth, the correlation between physical and mental health among the rural elderly has been neglected in previous studies.

### 2.3. The Framework and Hypotheses of this Study

**Figure 1:**
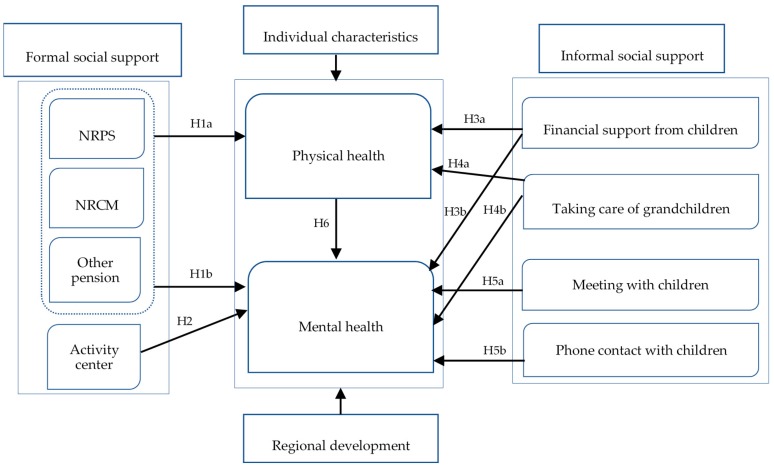
The framework of this study. NRPS, New Rural Pension Scheme; NRCM, New Rural Cooperative Medical.

There are two models for studying the effects of social support: a stress-buffering model and a direct effects model, although the measurements for social support are different between these types of studies [29,30,49]. The stress-buffering model argues that social support plays a moderating role and helps to improve physical and mental health by alleviating the pressure from liquidity constraints, loneliness, and anxiety, or by improving self-worth and self-fulfillment [49]. The direct effects model posits that social support affects health status independently, especially for mental health. For example, connecting with family members or friends can make the elderly happy [29]. We set the framework of this study by referring to these models, to previous studies, and to the social support system in China (Figure 1). In this framework, social support from the government and village collective is considered formal support because it is supported by national laws, policies, or public financing. Social support from family members is classified as an informal approach, since it is mainly dominated by morality and social opinion.

Formal support in China mainly refers to the social pension systems, the medical security systems, and the provisions of a special infrastructure for the rural elderly. The first two types of support aim to reduce liquidity constraints by increasing income and decreasing expenditures, respectively. However, health may be affected if liquidity constraints are reduced. For example, under this framework, the elderly are able to afford nutritious food and do not need to worry about lacking money. Furthermore, pensions are at the disposal of the elderly themselves, which is good for their self-esteem by providing ongoing financial support for the family. Therefore, the following hypotheses are proposed:

**Hypothesis** **1a.** *The NRPS, other pensions, and the NRCM have positive effects on the physical health of the rural elderly.*

**Hypothesis** **1b.** *The NRPS, other pensions, and the NRCM have positive effects on the mental health of the rural elderly.*

The provision of a rural elderly activity center aims to reduce elderly loneliness by supporting entertainment activities. For example, the rural elderly activity center provides chess for the elderly. Therefore, we hypothesize the following:

**Hypothesis** **2.** *The provision of an elderly activity center in the village has a positive effect on the metal health of the rural elderly.*

Most of the important informal social support in China comes from family members. Due to the limited amount of NRPS, financial support from children is very important for the rural elderly to reduce their liquidity constraints. Therefore, we hypothesize the following:

**Hypothesis** **3a.** *The effects of financial support from children on the physical health of the rural elderly are positive.*

**Hypothesis** **3b.** *The effects of financial support from children on the mental health of the rural elderly are positive.*

Furthermore, there are many left-behind children living with their grandparents in rural China. Taking care of grandchildren requires a great deal of energy, which may not be good for the elderly’s physical health. However, there is a tradition of enjoying family happiness (*tian lun zhi le*) in China. This means that the elderly enjoy spending time with their grandchildren, which makes them happy and less depressed. Therefore, the following hypotheses are proposed:

**Hypothesis** **4a.** *Taking care of their grandchildren has a negative effect on the physical health of the rural elderly.*

**Hypothesis** **4b.** *Taking care of their grandchildren has a positive effect on the mental health of the rural elderly.*

In China, many male members in family work outside the home and daughters rarely live with their parents after marriage. Therefore, meeting sons and daughters and communicating with them on the phone can ease loneliness of the elderly. Therefore, we hypothesize that:

**Hypothesis** **5a.** *Meeting with children has a positive effect on the mental health of the rural elderly.*

**Hypothesis** **5b.** *Phone contact with children has a positive effect on the mental health of the rural elderly.*

Generally, physical health status has an important effect on mental health. For example, the elderly with chronic diseases often worry about their illnesses, which makes them unhappy and depressed. Mental health also affects physical health, but this process is slow unless there is the serious depression that causes physical harm. Therefore, we hypothesize that:

**Hypothesis** **6.** *The healthier the body, the healthier the mind.*

## 3. Methods

### 3.1. Sampling and Data Collection

The data used in this study came from the China Rural Development Survey (CRDS) organized by the authors. The CRDS provides panel data and has been used in many other studies on rural human capital [50,51,52]. In each wave, the information was collected at the household and village levels of 100 villages in 25 counties across 5 provinces.

A stratified random sampling method was used to yield the sample in the first wave in 2005. First, each sample province was randomly selected from one of China’s major agro-ecological zones. According to the Chinese integrated regionalization of agriculture in 1981, there are nine agricultural regions in China, including agricultural and forestry areas in Northeast China; agricultural areas along Huanghuaihai; agricultural, forestry, and pastoral areas on the Loess Plateau; agricultural, forestry, and aquaculture areas in the Middle and Lower Reaches of the Yangtze River; agricultural and forestry areas in Southwest China; pastoral and forestry areas in Inner Mongolia and along the Great Wall; important agricultural and forestry farming areas in South China; pastoral and forestry areas in Gansu and Xinjiang; and pastoral areas on the Qinghai–Tibet Plateau. The first five are the major food-producing regions. We randomly selected one province in each major food-producing region, and thus five provinces were selected: Jiangsu, Sichuan, Shaanxi, Hebei, and Jilin.

Second, five sample counties were then selected from each province in a two-step procedure: (a) The enumeration team listed all the counties in each province in descending order of their per capita gross value of industrial output, which is a good predictor of the standard of living and development potential, and is often more reliable than net per capita income [53]; (b) the isometric random sampling method was used to select five counties per province from the resulting list.

Third, from each selected county, the team chose sample townships and villages according to their administrative divisions. Two townships were chosen from each county, with one from each of two groups per county: a “more well-off” group and a “poorer” group. Following the same procedure, two villages in each township were chosen.

Finally, the survey team randomly selected 20 households in each village. The enumeration team used the village roster that recorded each household’s *hukou* registration, including the name and contact number of the heads of the households in the village and the address of the household to randomly choose 20 households in each village.

In each household with elders aged above 60 years, we randomly selected one elder, who was living in the village, with a rural *hukou* to participate in a measurement of mental health in 2016. This method was employed to yield a nationally representative sample.

The survey collected information on individual and village characteristics by face-to-face interview. The individual characteristics included gender, age, years of schooling, party membership, and service as a village cadre. For the elderly aged above 60 years, we also gathered information about their social support, such as how frequently they met or contacted their children, their pension, whether they lived with their grandchildren, and the financial support received from their children. The information on informal social support and mental health was only collected in 2016. Information on village characteristics was also collected, such as whether the village provided the elderly with activity centers.

According to previous studies, self-reported physical health and depression indexes can be used as indicators to measure the physical and mental health of the rural elderly [11,54]. Consistent with previous studies, the self-reported physical health in this survey was measured by five ranks, namely, very good (5), good (4), common (3), bad (2), and very bad (1). There is relatively high agreement for physical health conditions when self-reported, compared to using a medical record review [55]. The depression index was measured in the survey using a short version of the depression scale from the Epidemiological Research Center (CES-D10), which has been used in previous studies [45,56,57,58]. We used Stata 13.0 to clean and analyze the data. The software of Stata 13.0 was developed by Stata company in Texas, TX, USA.

### 3.2. Definition of Variables

Physical health was measured by two methods: self-reported physical health with five ranks and a dummy variable constructed by self-reported health (1 = healthy, 0 = unhealthy). If the self-reported health of the elderly was “very good,” “good,” or “common,” we defined it as “healthy.” If the self-reported health was “bad” or “very bad,” we classified it as “unhealthy.” Mental health was measured by the elderly’s depression status (1 = depressed, 0 = undepressed) and depression score. CES-D10 involves 10 questions with a total score of 0–30 points to reflect the respondent’s mood and behavior in last week. If the score is 10 points or more, the individual is depressed. The higher the score, the deeper the depression.

This study divided social support into formal and informal support. Formal support was measured by three variables: (1) the amount of pension received each month in 2015 from the New Rural Pension Scheme (NRPS); (2) whether they had other kinds of pensions; and (3) whether they participated in the NRCM. These three variables were used to represent the social support from the government. The variable for whether there were elderly activity centers at the community level was used to measure the social support of the village collective.

Informal support included objective and subjective support. Financial support from children in 2015 was used to measure objective informal social support at the household level. The subjective informal support was measured by taking care of one’s grandchildren, the times of meeting with one’s children in 2015, and the number of phone calls with one’s children. In addition, individual characteristics of the health of the rural elderly were controlled to capture their effects. A description of these variables is shown in Table 1.

### 3.3. Model Specification

In order to systematically analyze the impact of social support on the health of the rural elderly, this study conducted a multiple regression analysis. First, Probit regression was adopted to identify the impact of social support on the physical and mental health of the rural elderly, while the dependent variables were the dummy variables. To conduct a robustness check of the determinants of physical health, we adopted ordered Probit regression using five ranks to measure physical health. The specifications are as follows:

Y_i_ = α + β_1_Formal_i_ + β_2_Informal_i_ + β_3_X_i_ + β_4_D_i_ + ε_i_ (1)

where i represents the i-th rural elderly; Y_i_ is the physical health or depression of the rural elderly in 2015; “Formal” indicates the formal support defined in Section 3.2; “Informal” indicates the informal support; X_i_ involves the individual characteristics, including gender, years of schooling, spouse status, currently or previously the village leader, and being a Communist Party of China (CPC) member; D_i_ indicates the dummy variables of the provinces; ε_i_ is the error term. β_1_, β_2_, β_3_, and β_4_ are the vectors of the estimated parameter, which describe the impact of formal support, informal support, and individual and regional characteristics on the physical and mental health of the elderly, respectively.

This study also focuses on the impact of social support on the degree of depression. Ordinary least squares (OLS) was used to estimate this effect. The specifications are as follows:

S_i_ = α + β_1_Formal_i_+ β_2_Informal_i_ + β_3_X_i_ + β_4_D_i_+ ε_i_ (2)

The dependent variable S in Equation (2) represents the depression score of the rural elderly. The definitions of the other variables and parameters are the same as those in Equation (1).

## 4. Descriptive Results

### 4.1. Physical and Mental Health of the Rural Elderly

A total of 774 rural elderly over 60 years old were used in this sample. The proportion of males was 64%, and the average age was 66.7. The average years of schooling was 5.2, and the proportion with a spouse was 81%. Seven percent of participants were or had currently or previously been the village cadre, while 15% were members of the Communist Party of China (CPC).

The data show that a large proportion of the rural elderly reported that they were unhealthy or suffering from depression (Table 2). The mean of self-reported physical health was 3.31, which is between “common” and “good.” Among the participants, 24.3% reported that they were unhealthy in their physical condition. The depression score was 7.74 on average, and 32.9% of the rural elderly suffered from depression. This result is similar to the results of previous studies [11,54]. These results suggest that the mental health of the rural elderly is worthy of attention, as well as their physical health.

There are significant regional differences in the physical and mental health of the rural elderly. In terms of physical health, 40% and 35.2% of the rural elderly were unhealthy in Sichuan and Shaanxi, respectively, which is far higher than the number in Jiangsu (12.9%), Jilin (13.5%), and Hebei (23.3%). In terms of mental health, the elderly also had a higher ratio of depression in Sichuan (47.4%) and Shaanxi (43.8%). The physical and mental health of the rural elderly in western provinces were worse (Table 2).

### 4.2. Social Support and Health of the Rural Elderly

Table 3 shows the relationship between social support and the physical and mental health among the rural elderly. In our sample, 35.9 % of the rural elderly were in receipt of a pension from NRPS, which is lower than the average amount (72.18 yuan a month). The physical and mental health of the rural elderly with NRPS pension less than the average amount are a little better than the health of those with NRPS pension more than the average amount. However, there was no significant difference between them (Table 3, rows 1 and 2). Only 19.6% of the rural elderly had another kind of pension; 84% of the rural elderly with another kind of pension reported that they were healthy, which is much higher than the score for those without another kind of pension (*p* < 0.05) (Table 3, rows 3 and 4, columns 3 and 4). The average depression score among the rural elderly with another kind of pension was 6.22, which is much lower than that of those without another kind of pension (*p* < 0.01) (Table 3, rows 3 and 4, columns 5 and 6). This indicates that the mental health of the elderly with another kind of pension is much better than the health of those receiving NRPS pensions or those without a pension.

Almost all of the rural elderly participated in the NRCM, which is the basic medical security system in rural China. There is no significant difference in the physical and mental health between the elderly with or without the NRCM (Table 3, rows 5 and 6). The community activity center was built for the entertainment of the elderly. The data show that there are no significant differences in the physical and mental health between the elderly in a village with a community activity center and those in a village without a community activity center (Table 3, rows 7 and 8).

The average financial support from children was 2784.57 yuan in 2015, which is about 3 times the pension from the NRPS. Although the elderly receiving financial support from their children above 2784.57 yuan had better physical and mental health than those receiving financial support from their children less than 2784.57 yuan, these differences are not statistically significant (Table 3, rows 9 and 10). The elderly who took care of their grandchildren had far better mental health than the elderly who did not care for their grandchildren (*p* < 0.05) (Table 3, rows 10 and 11, columns 5 and 6). The elderly who often met with their children had better health than those who rarely met with their children, but these differences are not statistically significant (Table 3, rows 13 and 14). The elderly who often connected with their children by phone were significantly healthier than those who rarely connected with their children by phone (*p* < 0.05) (Table 3, rows 15 and 16).

## 5. Multivariate Regression Results and Discussion

### 5.1. The Impact of Formal Social Support on the Health of the Rural Elderly

The results of the multivariable regression show that formal social support from the government and the village collective has no significant impact on the physical health of the rural elderly (Table 4, rows 1–4, columns 1–4). This finding indicates that H1a is not proven by our data, which implies that improving the physical health of the rural elderly should not only rely on providing formal social support, but must also further explore the emergency management of physical health and promote the utility of this formal support, such as medical examinations under the NRCM scheme.

The results show that formal social support has a significant impact on the mental health of the rural elderly. However, the direction and magnitude of these coefficients are different between different types of formal support. Specifically, the NRPS pension has no significant impact on the depression of the rural elderly, but it does have a significant effect on the depression score (*p* < 0.05) (Table 4, row 1, column 7). As the amount of the NRPS pension increases by 1 yuan in a month, depression score declines by 0.013 points on average. Having another pension insurance has positive effects on both depression and the depression score (*p* < 0.05) (Table 4, row 2, columns 6 and 7). This means that H1b is proven. If the elderly participate in other pension programs, they are 14.6% less likely to be depressed than the elderly who do not participate in other pension programs. The depression score of the elderly who participate in other pension programs is 2.223 lower than that of the elderly who do not participate in other pension programs. Participation in the NRCM has no effects on the mental health of the elderly (Table 4, row 3, columns 6 and 7). The presence of elderly activity centers has no effects on the mental health of the elderly (Table 4, row 4, columns 6 and 7). This finding indicates that H2 is not proven in this study.

### 5.2. The Impact of Informal Social Support on the Health of the Rural Elderly

Financial support from children, as the main objective support, has no effect on the elderly’s physical health (Table 4, row 5, columns 1–4). Thus, H3a is not proven. Financial support from children has no significant effect on the mental health of the rural elderly. Therefore, H3b is not proven.

Emotional support, a type of important informal support, has no effect on the physical health of rural elders. In particular, taking care of grandchildren does not worsen the elders’ physical health (Table 4, row 6, columns 1–4), which means that H4a is not proven. However, emotional support has a significant effect on the mental health of rural elders. Specifically, the rural elderly who take care of their grandchildren are 9.5% less likely to suffer depression than other elderly people that do not take care of their grandchildren (*p* < 0.01) (Table 4, row 6, columns 5–6). The depression score of the elders who take care of their grandchildren is 1.475 lower than those that do not take care of their grandchildren (*p* < 0.01) (Table 4, row 6, column 7). Thus, H4b is proven. The times of meeting with their children has no effect on the mental health of the rural elderly, which demonstrates that H5a is not proven in this study. The number of phone calls with children can significantly reduce the likelihood of depression among the rural elderly (*p* < 0.1) (Table 4, row 8, columns 5 and 6). Referring to the depression scores, if the number of phone calls with children increases by 1 time in a year, the elderly depression score decreases by 0.005 points on average (*p* < 0.01) (Table 4, row 8, column 7). These findings show that H5b is proven.

In addition, individual characteristics affect the health of the rural elderly. Consistent with the results of the descriptive analysis, males have better mental health than females (*p* < 0.05) (Table 4, row 9, columns 5–7). The more years of schooling they have, the better the physical health of rural elders (*p* < 0.01) (Table 4, row 11, columns 1–4). This means that the health return of education is significant among rural elders [59,60]. Being or having been the village cadre has significant effects on physical health (*p* < 0.05) (Table 4, row 13, columns 1–4).

Physical health also significantly affects the mental health of the elderly in rural areas. The possibility of being depressed is higher among the elders who are in poor physical health (*p* < 0.01) (Table 4, row 15, columns 5–7). Thus, H6 is proven. The income of elders with poor physical health may decrease while their medical expenditures increase, and they might require more care from others, which may increase their mental and material stress, and thus affect their mental health.

There is a significant regional disparity in the health of rural elders. The physical health of the rural elderly in the Sichuan, Shaanxi, and Hebei provinces is significantly worse than the health among elders in Jiangsu province, which is consistent with the descriptive analysis (Table 4, rows 15, 17 and 19, columns 1–4). The rural elderly in western China are more likely to be depressed (Table 4, rows 16 and 17, columns 5 and 6). Compared with Jiangsu, which is located on the east coast and is one of the most developed provinces in China, Sichuan and Shaanxi, located in western China, are very undeveloped. The huge gap in economic and social development, such as public infrastructure and services, results in a significant difference in health status.

### 5.3. Discussion

The results show that the NRPS and other pensions have different effects on mental health among rural elders. The NRPS pension is limited in rural China and is not enough to significantly reduce the likelihood of depression among rural elders. However, it could significantly relieve the degree of depression among the rural elderly. Other pensions, such as those provided by commercial pension insurance, offer a large pension amount, which could alleviate liquidity constraints. This could not only significantly reduce the likelihood of depression among the elderly, but could also effectively reduce their depression score. In addition, large pensions might increase the self-esteem of the elderly, because such pensions will help them to provide economic support for their family rather than to take money from their children, which may help decrease their degree of depression.

Taking care of grandchildren has a positive effect on the mental health of rural elders, which is consistent with the results of previous studies [61]. In traditional Chinese culture, people like the happiness associated with family members (*tian lun zhi le*), especially elders. In addition, taking care of their grandchildren generally helps the rural elderly increase their self-esteem by supporting their family rather than just receiving help from them. A previous study found that the elderly who provided assistance to others more often rated their health more favorably than those who were less involved in helping others [62]. The number of phone calls with their children in a year also has a positive effect on mental health, but the number of meetings with their children has no effect. There may be two reasons for this result, but they cannot be proven due to data limitations: (a) Rural elders usually live with their son(s) together or in the same village. Thus, they meet with their son(s) much more frequently than with their daughter(s), but son(s) are not as good as daughter(s) at emotionally communicating with their parents. (b) In rural China, elders rarely live with their daughter(s) after their daughter(s) get married. Therefore, a daughter is more likely to communicate with an elderly family member by phone. Since daughters are good at emotionally communicating with their parents, the number of phone calls with children in a year has a positive effect on mental health. There is a folk adage in China that a daughter is her parents’ little cotton-padded jacket, which means that communicating with their daughter(s) makes elderly family members happier than communicating with their son(s).

Comparing material support with formal support and informal support, we find that the financial support provided by children does not have a significant impact on the mental health of the rural elderly, while an increase in the amount of pension does significantly inhibit the possibility and degree of depression. The reason for this result might be that pension income is more stable than the financial support of children. Higher pensions could therefore reduce the elderly’s uncertainty about the future, effectively reducing poverty, relieving the pressure of life, and improving and promoting satisfaction with life [63,64]. This finding is consistent with the results of previous studies [14]. In addition, compared with material support, emotional support has a greater effect on promoting rural elders’ mental health (such as the company of grandchildren and phone calls with their children).

## 6. Conclusions

This paper used nationally representative micro-survey data on the rural elderly aged over 60 to analyze the status and impact of physical and mental health among them for the year 2015. In particular, this study estimated the impacts of different types of social support on the physical and mental health of the rural elderly using Probit, Oprobit, and OLS regression. According to this study, we conclude the following:

First, the outlook for the physical and mental health of the rural elderly is not good, especially for females. A total of 24.3% of the rural elderly in the 2015 sample had physical health problems, and 32.9% of them were depressed. The physical and mental health among the elderly in the western provinces was even worse.

Second, social support does not promote the physical health of the elderly in rural areas, but it has a significant positive impact on their mental health, especially as it relates to emotional support. Specifically, a pension from the NRPS or other kinds of pensions, taking care of grandchildren, and phone contact with children can effectively alleviate the depression of the rural elderly.

Based on our results, the following policy and academic suggestions are proposed to help achieve the universal health of residents and to narrow the health gap between urban and rural areas.

First, the government should pay more attention to the mental health of the rural elderly when focusing on their physical health, especially the female rural elderly and those in western provinces. The government should take the following measures: (a) setting mental health services for the rural elderly as one item for current public health services in rural areas; (b) investigating the mental health of the rural elderly by regularly conducting psychological testing; and (c) increasing the amount of the NRPS pension to ease the liquidity constraints of the rural elderly.

Second, the government should encourage and support emotional communication for the rural elderly. On the one hand, the government develop traditional Chinese culture to encourage family members, such as children and grandchildren, to provide more emotional support. On the other hand, it is necessary to adopt other approaches to provide emotional support for rural elderly, such as providing free mobile phones for the economically disadvantaged elderly and teaching them how to use them. This would be helpful for the mental health of both the elderly who live alone and their family members.

Third, elderly activity centers should be promoted. The government spends a great deal of money building rural elderly activity centers, and the village collectives spend a great deal of time managing them. However, this does not seem to have the desired effect. Specific activities, utilization, and their effects on the mental health of the rural elderly should be focused on in the future.

It should be noted that this study conducted a correlation analysis with a relatively small sample. The robust causal relationship, revealed by multiple econometric models, between social support and the rural elderly’s physical and mental health is still worthy of further research. It is also very important to conduct further studies based on a large sample with much richer information to explore the mechanisms underlying the effects of social support on the elderly health. Quantitative data and scales are needed to measure rural social support and physical health in order to precisely determine the relationship between them.

## Figures and Tables

**Table 1:** Descriptive statistics of variables (*N* = 774).

Variable	Description	Mean	Standard Deviation	Min	Max
Amount of pension from the NRPS	Yuan per month	72.18	49.99	0	300
Do you have any other pension?	1 = Yes, 0 = No	0.19	0.39	0	1
Do you participate in the NRCM?	1 = Yes, 0 = No	0.97	0.15	0	1
Is there an elderly activity center in the village?	1 = Yes, 0 = No	0.14	0.35	0	1
Amount of financial support from children	Yuan per year	2784.57	4690.79	0	40,000
Taking care of grandchildren	1 = Yes, 0 = No	0.31	0.46	0	1
Times you met with your children in 2015	Times	166.57	166.98	0.07	365
Number of phone calls with children in 2015	Times	78.28	116.78	0.06	365
Gender	1 = Male, 0 = Female	0.64	0.48	0	1
Age	Years	66.72	5.20	60	87
Years of schooling	Years	5.20	3.51	0	16
Do you have a spouse?	1 = Yes, 0 = No	0.81	0.39	0	1
Currently or previously the village leader	1 = Yes, 0 = No	0.07	0.26	0	1
Being a CPC member	1 = Yes, 0 = No	0.15	0.36	0	1

Note: For the elderly living with children, the times of meeting and phone calls with children was 365 in 2015. Data source: China Rural Development Survey (CRDS). CPC, Communist Party of China.

**Table 2:** Physical and mental health of the rural elderly.

Province	Self-Reported Physical Health			Mental Health		
	Mean of Five Ranks	Unhealthy (%)	Healthy (%)	Mean of Depression Score	Depression (%)	Non-Depression (%)
Jiangsu	3.72	12.9	87.1	6.12	22.2	77.8
Sichuan	2.85	40.0	60.0	10.29	47.4	52.6
Shaanxi	2.87	35.2	64.8	9.39	43.8	56.2
Jilin	3.59	13.5	86.5	6.63	26.2	73.8
Hebei	3.36	23.3	76.7	6.75	28.9	71.1
Total	3.31	24.3	75.7	7.74	32.9	67.1

Data source: China Rural Development Survey (CRDS).

**Table 3:** Self-reported physical health and depression scores with different characteristics.

	Variable	*N*	%	Self-Reported Physical Health	t-Value	Depression Score	t-Value
		(1)	(2)	(3)	(4)	(5)	(6)
	Amount of pension from the NRPS						
	
(1)	≤Average value	278	35.9	0.76	−0.266	7.57	0.523
(2)	>Average value	496	64.1	0.75		7.83	
	Do you have any other pension?						
(3)	Yes	152	19.6	0.84	−2.522 **	6.22	3.141 ***
(4)	No	622	80.4	0.74		8.11	
	Do you participate in the NRCM?						
(5)	Yes	756	97.7	0.76	−0.349	7.75	−0.296
(6)	No	18	2.3	0.72		7.28	
	Is there an elderly activity center in the village?						
(7)	Yes	108	13.9	0.81	−1.265	6.95	1.315
(8)	No	666	86.1	0.75		7.86	
	Amount of financial support from children						
(9)	≤Average value	559	72.2	0.74	1.539	7.90	−1.091
(10)	>Average value	215	27.8	0.80		7.32	
	Taking care of grandchildren						
(11)	Yes	241	31.1	0.75	0.445	6.97	2.151 **
(12)	No	533	68.9	0.76		8.09	
	Times you met with your children in 2015						
(13)	≤Average value	439	56.7	0.74	−1.077	7.82	0.400
(14)	>Average value	335	43.3	0.78		7.63	
	Number of phone calls with children in 2015						
(15)	≤Average value	554	88.2	0.74	2.129 **	8.39	−4.312 ***
(16)	>Average value	220	11.8	0.81		6.11	

Note: *** *p* < 0.01, ** *p* < 0.05. Data source: China Rural Development Survey (CRDS).

**Table 4:** The effects of social support on the physical and mental health of the rural elderly.

	Variables	Self-Assessment of Physical Health				Mental Health		
		Probit (1 = Healthy; 0 = Unhealthy)	dy/dx	Oprobit (1 = Very Bad, 2 = Bad, 3 = Common, 4 = Good, 5 = Very Good)	dy/dx	Depression (1 = yes; 0 = no)	dy/dx	Depression Score
		(1)	(2)	(3)	(4)	(5)	(6)	(7)
	Formal support							
(1)	Amount of pension from the NRPS	0.001	0.000	0.001	−0.000	−0.002	−0.001	−0.013 **
		(0.002)	(0.000)	(0.001)	(0.000)	(0.002)	(0.001)	(0.006)
(2)	Do you have any other pension?	0.251	0.070	0.147	−0.013	−0.468 **	−0.146 **	−2.223 ***
		(0.213)	(0.060)	(0.152)	(0.014)	(0.200)	(0.062)	(0.810)
(3)	Do you participate in the NRCM?	0.424	0.119	0.116	−0.010	0.037	0.011	−0.189
		(0.350)	(0.098)	(0.256)	(0.023)	(0.349)	(0.109)	(1.228)
(4)	Is there an elderly activity center in the village?	0.088	0.025	−0.110	0.010	0.030	0.009	−0.032
		(0.163)	(0.046)	(0.114)	(0.010)	(0.152)	(0.047)	(0.593)
	Informal support							
(5)	Amount of financial support from children	−0.000	−0.000	−0.000	0.000	−0.000	−0.000	−0.000
		(0.000)	(0.000)	(0.000)	(0.000)	(0.000)	(0.000)	(0.000)
(6)	Taking care of grandchildren	−0.025	−0.007	0.080	−0.007	−0.305 ***	−0.095 ***	−1.475 ***
		(0.119)	(0.033)	(0.088)	(0.008)	(0.117)	(0.036)	(0.480)
(7)	Times you met with your children in 2015	0.000	0.000	0.000	−0.000	0.000	0.000	0.001
		(0.000)	(0.000)	(0.000)	(0.000)	(0.000)	(0.000)	(0.001)
(8)	Number of phone calls with children in 2015	0.000	0.000	0.000	−0.000	−0.001 *	−0.000*	−0.005 ***
		(0.001)	(0.000)	(0.000)	(0.000)	(0.000)	(0.000)	(0.002)
	Individual characteristics							
(9)	Gender	0.111	0.031	0.037	−0.003	−0.260 **	−0.081 **	−1.235 **
		(0.120)	(0.034)	(0.091)	(0.008)	(0.116)	(0.036)	(0.534)
(10)	Age	0.002	0.001	−0.009	0.001	−0.011	−0.003	−0.025
		(0.011)	(0.003)	(0.008)	(0.001)	(0.011)	(0.003)	(0.044)
(11)	Years of schooling	0.051 ***	0.014 ***	0.040 ***	−0.004 ***	−0.001	−0.000	−0.073
		(0.017)	(0.005)	(0.013)	(0.001)	(0.017)	(0.005)	(0.073)
(12)	Do you have a spouse?	−0.179	−0.050	−0.201 **	0.018 *	−0.120	−0.037	−0.898
		(0.138)	(0.039)	(0.102)	(0.009)	(0.131)	(0.041)	(0.615)
(13)	Currently or previously the village leader	0.518 **	0.145 **	0.275 *	−0.025 *	0.065	0.020	−0.437
		(0.258)	(0.072)	(0.155)	(0.014)	(0.205)	(0.064)	(0.734)
(14)	Being a CPC member	0.258	0.073	0.090	−0.008	−0.153	−0.048	−0.735
		(0.164)	(0.046)	(0.111)	(0.010)	(0.153)	(0.048)	(0.547)
(15)	Physically healthy (1 = yes; 0 = no)					−0.803 ***	−0.251 ***	−4.919 ***
						(0.116)	(0.033)	(0.596)
(16)	Sichuan Province	−0.763 ***	−0.222 ***	−0.752 ***	0.063 ***	0.414 **	0.135 **	1.832 ***
		(0.169)	(0.048)	(0.124)	(0.014)	(0.163)	(0.053)	(0.689)
(17)	Shaanxi Province	−0.666 ***	−0.188 ***	−0.779 ***	0.067 ***	0.323 *	0.104 *	1.100
		(0.182)	(0.053)	(0.135)	(0.017)	(0.175)	(0.057)	(0.758)
(18)	Jilin Province	−0.106	−0.024	−0.150	0.007	0.118	0.036	0.279
		(0.194)	(0.044)	(0.128)	(0.006)	(0.173)	(0.053)	(0.692)
(19)	Hebei Province	−0.387 **	−0.098 **	−0.321 ***	0.017 **	−0.049	−0.015	−0.873
		(0.173)	(0.044)	(0.121)	(0.008)	(0.164)	(0.048)	(0.649)
(20)	Constant	0.174				1.327		17.264 ***
		(0.849)				(0.834)		(3.415)
(21)	(Pseudo) R^2^	0.101		0.05		0.128		0.227
(22)	*N*	774	774	774	774	774		774

Note: *** *p* < 0.01, ** *p* < 0.05, * *p* < 0.1. Standard error is reported in parentheses. Column 2 is the marginal effect of the Probit regression on self-reported physical health. Column 4 is the marginal effect of the Oprobit regression. Column 6 is the marginal effect of Probit regression on mental health. The number of individuals of very bad, bad, common, good, and very good in physical health are 36, 152, 250, 208, and 128, respectively. The cut1, cut2, cut3, and cut4 are omitted; they are −2.391, −1.324, −0.337, and 0.496, respectively. Data source: China Rural Development Survey (CRDS).
